# Prediction of mortality and functional outcome from status epilepticus and independent external validation of STESS and EMSE scores

**DOI:** 10.1186/s13054-016-1190-z

**Published:** 2016-01-27

**Authors:** Bong Su Kang, Dong Wook Kim, Kwang Ki Kim, Hye Jin Moon, Young-Soo Kim, Hyun Kyung Kim, Seo-Young Lee, Yong Seo Koo, Jung-Won Shin, Jangsup Moon, Jun-Sang Sunwoo, Jung-Ick Byun, Yong Won Cho, Ki-Young Jung, Kon Chu, Sang Kun Lee

**Affiliations:** 1 Department of Neurology, Korea University Anam Hospital, Seoul, South Korea; 2 Department of Neurology, Konkuk University School of Meidcine, Seoul, South Korea; 3 Department of Neurology, Dongguk University Ilsan Hospital, Goyang, South Korea; 4 Department of Neurology, Dongsan Medical Center, Keimyung University, Daegu, South Korea; 5 Department of Neurology, Samsung Changwon Hospital, Sungkyunkwan University School of Medicine, Changwon, South Korea; 6 Department of Neurology, National Medical Center, Seoul, South Korea; 7 Department of Neurology, Kangwon National University School of Medicine, Chuncheon, South Korea; 8 Department of Neurology, CHA Bundang Medical Center, CHA University, Seongnam, South Korea; 9 Department of Neurology, Laboratory for Neurotherapeutics, Comprehensive Epilepsy Center, Biomedical Research Institute, Seoul National University Hospital, College of Medicine, Seoul National University, Seoul, South Korea

**Keywords:** Status epilepticus, Functional outcome, Prediction model, STESS, EMSE

## Abstract

**Background:**

Two clinical scoring systems, the status epilepticus severity score (STESS) and the epidemiology-based mortality score in status epilepticus (EMSE), are used to predict mortality in patients with status epilepticus (SE). The aim of this study was to compare the outcome-prediction function of the two scoring systems regarding in-hospital mortality using a multicenter large cohort of adult patients with SE. Moreover, we studied the potential role of these two scoring systems in predicting the functional outcome in patients with SE.

**Methods:**

The SE cohort consisted of patients from the epilepsy centers of eight academic tertiary medical centers in South Korea. The clinical and electroencephalography data for all adult patients with SE from January 2013 to December 2014 were derived from a prospective SE database. The primary outcome variable was defined as in-hospital death. The secondary outcome variable was defined as a poor functional outcome, i.e., a score of 1–3 on the Glasgow Outcome Scale, at discharge.

**Results:**

Among the 120 non-hypoxic patients with SE recruited into the study, 16 (13.3 %) died in the hospital and 64 (53.3 %) were discharged with a poor functional outcome. The receiver-operating characteristic (ROC) curve for prediction of in-hospital death based on the STESS had an area under the curve of 0.673 with an optimal cutoff value for discrimination (best match for both sensitivity (0.56) and specificity (0.70)) that was ≥4 points. The two combinations of elements of the EMSE system (EMSE-ALDEg and EMSE-ECLEg) predicted not only in-hospital mortality with the best match for sensitivity (more than 0.6) and specificity (more than 0.6), but also a poor functional outcome with the best match for both sensitivity (>0.7) and specificity (>0.6). STESS did not predict a poor functional outcome (area under the ROC, 0.581; *P =* 0.23).

**Conclusion:**

Although the EMSE is a clinical scoring system that focuses on individual mortality, we did not find differences between the EMSE and STESS in the prediction of in-hospital death. The EMSE was useful in predicting poor functional outcome, as it was significantly better than STESS.

**Electronic supplementary material:**

The online version of this article (doi:10.1186/s13054-016-1190-z) contains supplementary material, which is available to authorized users.

## Background

Status epilepticus (SE) is a common neurological emergency that is associated with high rates of mortality and morbidity. Thus, rapid initiation of treatment tailored to the severity of SE is required. In this regard, prediction of bad outcome is needed to avoid underdetection and undertreatment of SE. Adequate prediction of good outcome is crucial to minimize risks of potentially harmful overtreatment. Two clinical scoring systems, the status epilepticus severity score (STESS) [1] and the epidemiology-based mortality score in status epilepticus (EMSE) [2], are available to predict the risk of death at SE onset.

The STESS consists of four clinical parameters, including level of consciousness, worst seizure type, age, and history of previous seizure [1]. Validation studies have reported that a score of ≥3 points on the STESS indicates a poor outcome [1, 3]; in contrast, a recent study reported a different optimized cutoff value for survival versus death (≥4 points) [4]. The EMSE is another clinical scoring system that is used for outcome prediction in patients with SE [2]. It was published with both three (etiology, age, comorbidity; EAC) and four (etiology, age, comorbidity, electroencephalography (EEG); EACE) parameters, and scores “mortality risk points”. It has been reported that the combination of EMSE-EAC and EMSE-EACE is superior to STESS in explaining individual mortality in SE; however, no external validation study of this issue has been performed.

Disability among survivors and return to the patients’ premorbid state may also be very interesting and important outcome parameters. However, the two clinical scoring systems were not initially tested for prediction of individual functional outcome [1, 2]. The aim of this study was to compare the outcome-prediction functions of the STESS and EMSE regarding in-hospital mortality in a large prospective cohort of adult patients with SE. Additionally, we studied the potential role of these two scoring systems in predicting the functional outcome in patients with SE.

## Methods

### Setting

This study was performed in the epilepsy centers of eight academic tertiary hospitals in South Korea. The clinical and EEG data of all adult patients with SE from January 2013 to December 2014 were derived from a prospective SE database. The study was approved by the institutional review boards of the lead study center (Korea University Anam Hospital, Seoul, South Korea; IRB no. ED-13157) and all other participating hospitals (see Additional file 1) in accordance with the standards of the Declaration of Helsinki. Informed consent was waived.

### Definition of SE

SE was defined as the presence of clinical and EEG evidence of seizures lasting at least 5 min or of repeated seizures without intervening recovery of consciousness [5]. The types of SE were classified according to the initial manifestation as: (1) generalized convulsive SE (GCSE) and (2) non-convulsive SE (NCSE). If SE began with a generalized tonic–clonic seizure with continuous convulsion or evolution to a non-convulsive status, it was defined as GCSE. NSCE was defined as a condition with prolonged electrographic seizure activity resulting in non-convulsive behavioral and/or cognitive changes from the baseline [6]. Refractory SE was defined as SE that was unresponsive to one adequate dose of benzodiazepine and one antiepileptic drug.

### Data collection and scoring of SE

**Fig. 1:**
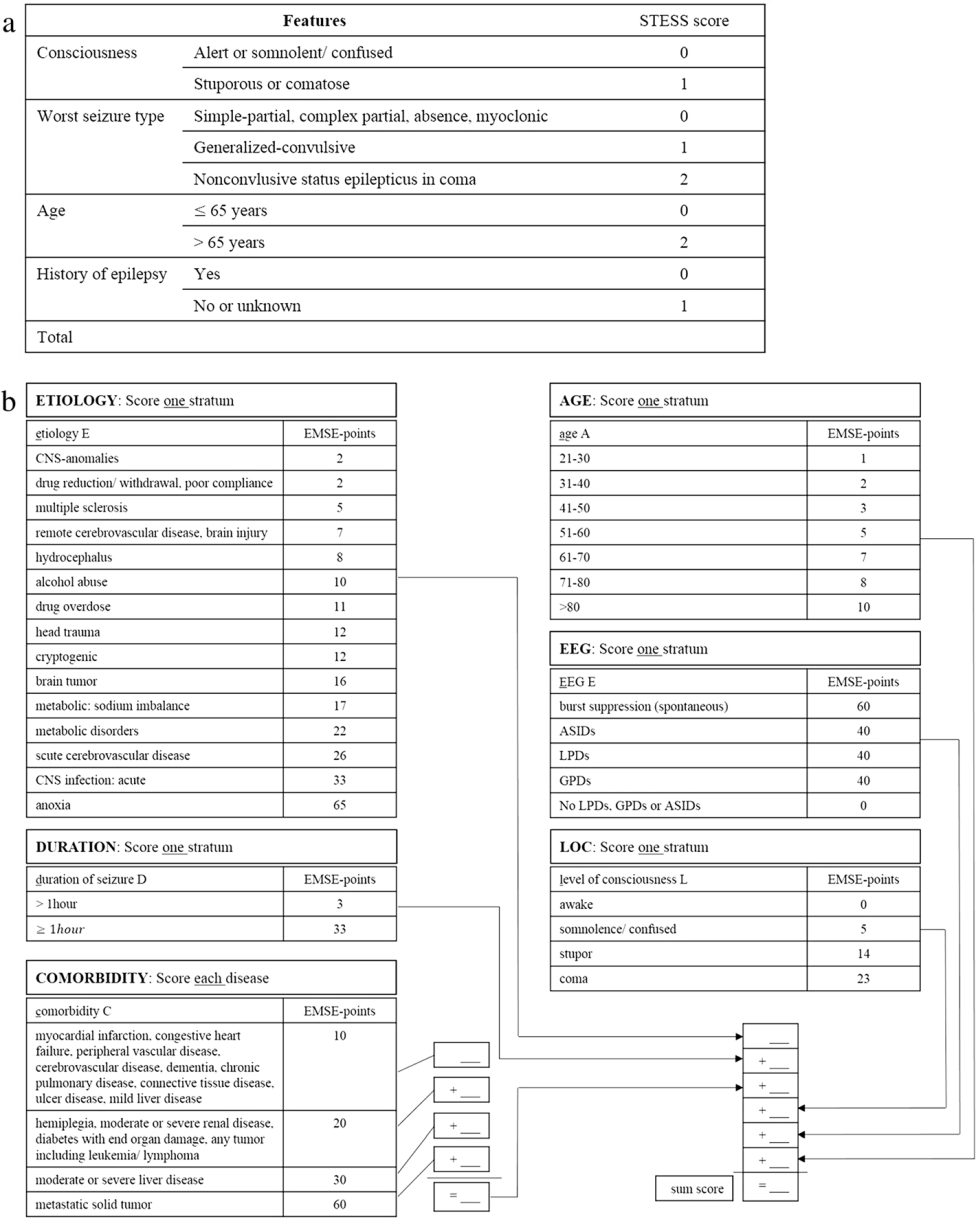
Evaluation example for **a** STESS and **b** EMSE systems. *ASID after status ictal discharge*, *CNS* central nervous system, *EEG* electroencephalography, *EMSE* epidemiology-based mortality score in status epilepticus, *GPD* generalized periodic discharge, *LOC* level of consciousness, *LPD* lateralized periodic discharge, *STESS* status epilepticus severity score

Information was collected regarding the clinical and EEG features at SE onset. The data included all integral components of the STESS and EMSE, i.e., age, etiology of SE (categorized as proposed by the International League Against Epilepsy (ILAE)) [7], history of epilepsy, worst seizure type, level of consciousness (LOC) before treatment, seizure duration, initial EEG pattern, and comorbidity, and were determined at SE onset. The STESS was calculated as reported previously (Fig. 1a) [1]. Moreover, the mortality risk points of six EMSE parameters in the fields of etiology, age, LOC, seizure duration, EEG pattern, and comorbidity were collected, and the sum score of all possible combinations of EMSE elements was calculated as reported previously (Fig. 1b) [2].

### Outcome

The primary outcome variable was defined as death in the hospital, which also included patients who were transferred to a palliative care unit when death was expected in the near future. The secondary outcome variable was defined as a poor functional outcome, i.e., a score of 1–3 on the Glasgow Outcome Scale (GOS), at discharge.

### Statistics

Statistical analyses were performed using the SPSS 20.0KO software for Windows. Categorized variables were summarized as counts and proportions, and continuous variables were summarized as median value and range. The categorical variables were compared using Fisher’s exact test or the chi-squared test, and continuous categorical variables were compared using Student’s *t*-test or the Mann–Whitney *U* test. *P* values ≤0.05 were considered statistically significant.

We defined discrimination as the ability of the STESS and EMSE to segregate survivors from non-survivors and to differentiate patients who had a good functional outcome from patients who had a poor functional outcome. This was quantified by calculating the c static analogous to the area under a receiver-operating characteristic curve (AUROC) [8], which estimated the probability that a model assigned a higher risk of in-hospital mortality or a poor functional outcome to those who had a high score on each scoring system compared with those with a low score on each system. Youden’s index was calculated to identify the optimal cutoff point of each score regarding sensitivity and specificity for the prediction of outcomes. Sensitivity, specificity, positive and negative predictive value (PPV and NPV, respectively), and accuracy were calculated for the EMSE and STESS.

To determine the optimal combination of EMSE elements we used a two-step approach. First, we chose EMSE combinations for in-hospital death that (1) had a higher AUROC than the AUROC of the STESS, (2) had a high sensitivity (>0.6) and specificity (>0.6) at the calculated cutoff point, and (3) combined less than four parameters. Next, we analyzed the predicting power of choosing an EMSE combination for functional outcome, AUROC, and calculated cutoff point. The EMSE combination that could simultaneously predict in-hospital mortality and functional outcome and had high sensitivity and specificity was considered the optimal EMSE combination.

## Results

### Demographics

One hundred twenty non-hypoxic patients with SE from eight centers (3–24 patients from each center) were enrolled in this study. The demographics and clinical characteristics of the cohort are summarized in Table 1. Among the 120 patients, 16 (13.3 %) died in the hospital and 64 (53.3 %) were discharged with a poor functional outcome. Refractory SE (22.1 % of survivors versus 56.2 % of non-survivors; *P* = 0.007), a stuporous or comatose mental status at pretreatment (48.1 % of survivors versus 87.5 % of non-survivors; *P* = 0.003), and the presence of periodic discharge at the initial EEG (50.0 % of survivors versus 81.2 % of non-survivors; *P* = 0.029) were associated with in-hospital mortality. Older age (a median of 54 years for a good functional outcome versus 67 years for a poor functional outcome; *P* = 0.036), refractory SE (15.1 % for a good versus 35.8 % for a poor functional outcome; *P* = 0.011), seizure duration over 1 hour (64.2 % for a good versus 80.6 % for a poor functional outcome; *P* = 0.043), the presence of burst suppression (9.4 % for a good versus 26.9 % for a poor functional outcome; *P* = 0.016), and the presence of periodic discharge at the initial EEG (32.1 % for a good versus 71.6 % for a poor functional outcome; *P* < 0.0001) were associated with a poor functional outcome at discharge.

**Table 1:** Demographic and clinical characteristics of the patients

	Total (n = 120)
Gender (female, %)	56 (46.7 %)
Age (years, median (range))	63.5 (20–91)
Age (>65 years)	57 (47.5 %)
Etiology of SE	
Acute symptomatic	50 (41.7 %)
Remote symptomatic	44 (36.7 %)
Progressive symptomatic	9 (7.5 %)
Idiopathic/cryptogenic	17 (14.2 %)
SE dynamics	
Generalized convulsive SE only	39 (32.5 %)
Non-convulsive SE	50 (41.7 %)
Generalized convulsive SE evolving into non-convulsive SE	19 (15.8 %)
Absence SE	1 (0.8 %)
First episode of SE	106 (88 %)
Refractory SE	28 (23.3 %)
LOC pretreatment	
Awake or somnolent/confused	56 (46.7 %)
Stuporous or comatose	64 (53.3 %)
Worst seizure type	
Simple partial, CPS, absence, myoclonic	48 (40.0 %)
Generalized convulsive	48 (40.0 %)
Non-convulsive SE in coma	24 (20.0 %)
History of previous epilepsy	77 (64.2 %)
Duration of pretreatment (>1 hour)	88 (73.3 %)
Duration of seizure	
>5 min, <30 min	10 (8.3 %)
3 min or longer, but <1 hour	15 (12.5 %)
1 hour or longer	95 (79.2 %)
Charlon’s Comorbidity index total	
0	33 (27.5 %)
1–2	41 (34.1 %)
3 or more	46 (38.4 %)
EEG–BS	23 (19.2 %)
PDs	65 (54.2 %)
Treated on NICU	57 (47.5 %)
Days on NICU (median (range))	8 (1–125)

Values are given as n (%) unless otherwise indicated. *BS* burst suppression, *CPS* complex partial seizure, *EEG* electroencephalography, *LOC* level of consciousness, *NICU* neurological intensive care unit, *PD* periodic discharge, *SE* status epilepticus

### Performance of the STESS and EMSE in predicting in-hospital death

**Fig. 2:**
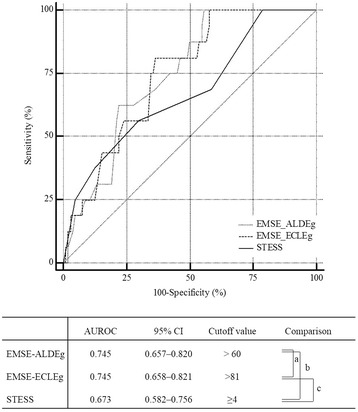
Receiver-operating characteristic curves for EMSE-ALDEg, ECLEg and STESS for predicting in-hospital mortality. Comparison *P* value: a = 0.9928, b = 0.3205, c = 0.4591. *AUROC* area under a receiver-operating characteristic curve, *CI* confidence interval, *EMSE* epidemiology-based mortality score in status epilepticus (E, etiology; A, age; C, comorbidity; L, level of consciousness at pretreatment; D, duration; Eg, electroencephalography), *STESS* status epilepticus severity score

The receiver-operating characteristic (ROC) curve for predicting in-hospital death based on the STESS had an area under the curve (AUC) of 0.673 with an optimal cutoff value for discrimination (best match for both sensitivity (0.56) and specificity (0.70)) that was ≥4 points. Twenty-eight combinations of the EMSE (3 combinations with 2 domains, 12 with 3 domains, 8 with 4 domains, and all combinations with 5 or 6 domains) had an AUROC >0.700 (Additional file 2: Table S1). Among the eight EMSE combinations that had the highest AUROC, EMSE-ALDEg (age-loss of consciousness-duration-EEG) and EMSE-ECLEg (etiology-comorbidity-loss of consciousness-EEG) were the combinations that exhibited the best match for sensitivity (>0.6) and specificity (>0.6) above their calculated cutoff values (Table 2). There was no statistically significant difference between the ROC for predicting in-hospital death based on the EMSE-ALDEg and EMSE-ECLEg, and the ROC for predicting in-hospital death based on the STESS (Fig. 2).

**Table 2:** Quantitative description of the choice of EMSE combinations and STESS for predicting death

	AUROC (cutoff point)	Sensitivity	Specificity	NPV	PPV	Accuracy
EMSE-EAC	0.633 (>37)	0.17	0.92	0.47	0.75	0.51
EMSE-ECEg	0.750 (>71)	0.81	0.56	0.95	0.22	0.59
EMSE-ELEg	0.764 (>47)	1.00	0.46	1.00	0.22	0.53
EMSE-LDEg	0.744 (>47)	0.21	1.00	0.40	1.00	0.48
EMSE-EACEg	0.712 (>62)	0.94	0.44	0.98	0.21	0.51
EMSE-EALEg	0.771 (>54)	1.00	0.47	1.00	0.22	0.54
EMSE-ECLEg	0.745 (>81)	0.81	0.63	0.96	0.25	0.66
EMSE-ELDEg	0.766 (>75)	0.23	1.00	0.47	1.00	0.59
EMSE-ALDEg	0.745 (>60)	0.22	1.00	0.44	1.00	0.52
STESS	0.673 (≥4)	0.56	0.70	0.91	0.22	0.68

*AUROC* area under a receiver-operating characteristic curve, *EMSE* epidemiology-based mortality score in status epilepticus (E, etiology; A, age; C, comorbidity; L, level of consciousness at pretreatment; D, duration; Eg, electroencephalography), *NPV* negative predictive value, *PPV* positive predictive value, *STESS* status epilepticus severity score

**Fig. 3:**
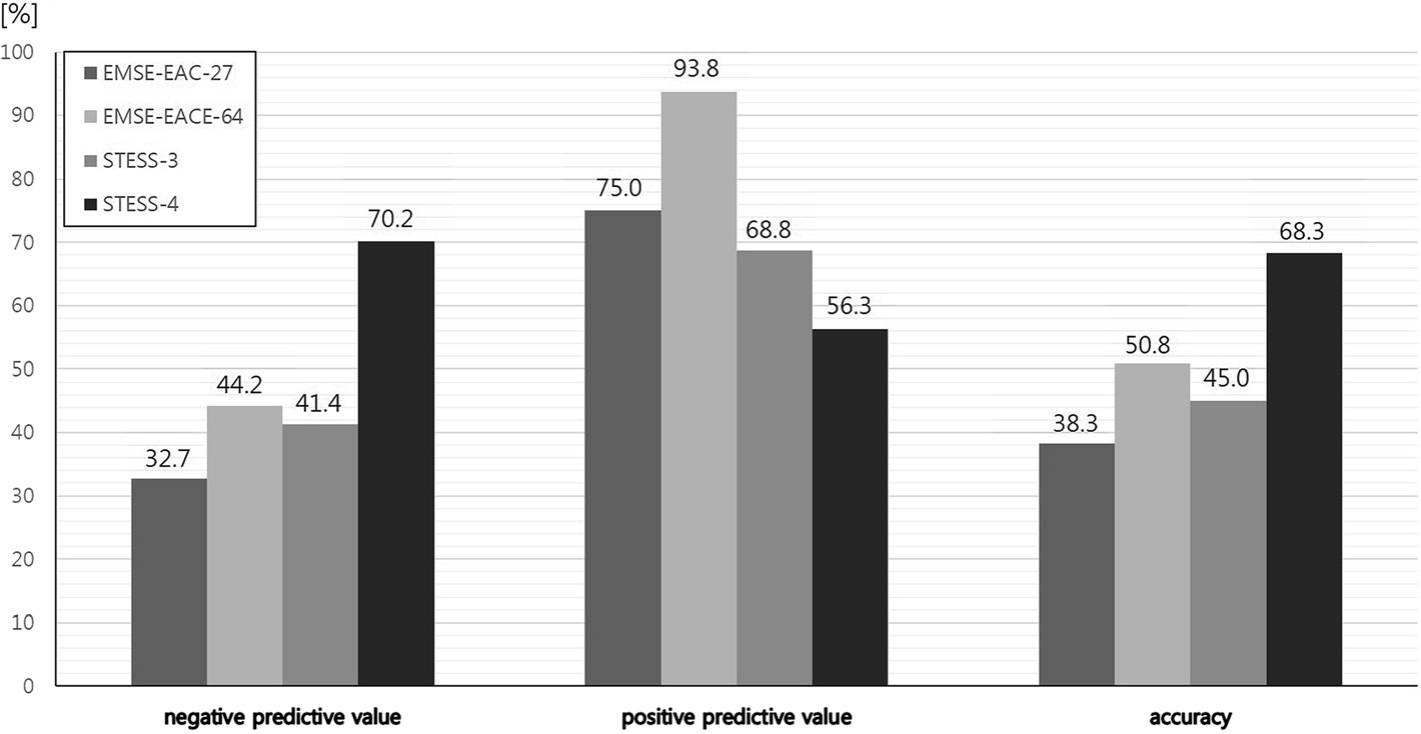
Comparison of test performances between epidemiology-based mortality score in status epilepticus (*EMSE*) etiology–age–comorbidity (*EAC*)-27, etiology–age–comorbidity–EEG (*EACE*)-65, status epilepticus severity score (*STESS*)-3 and STESS-4. Negative predictive value (*NPV*): *P* = 0.0323 for EAC versus EACE, *P* = 0.0141 for EAC versus STESS-3, *P* = 0.8672 for EAC versus STESS-4, *P* = 0.1633 for EACE versus STESS-3, *P* = 0.2221 for EACE versus STESS-4, *P* = 0.342 for STESS-3 versus STESS-4; positive predictive value (*PPV*): *P* = 0.0192 for EAC versus EACE, *P* = 0.1185 for EAC versus STESS-3, *P* = 0.2268 for EAC versus STESS-4, *P* = 0.0063 for EACE versus STESS-3, *P* = 0.0215 for EACE versus STESS-4, *P* = 0.424 for STESS-3 versus STESS-4; accuracy: *P* = 0.3017 for EAC versus EACE, *P* = 0.0927 for EAC versus STESS-3, *P* = 0.5753 for EAC versus STESS-4, *P* = 0.7069 for EACE versus STESS-3, *P* = 0.0635 for EACE versus STESS-4, *P* = 0.2174 for STESS-3 versus STESS-4; level of significance corrected for multiple testing *P* ≤ 0.0044

There were no significant differences between the test performance of EMSE-EAC and EMSE-EACE with initial published cutoff, EAC-27 and EACE-64 and those of STESS-3 and STESS-4 (Fig. 3). The lowest total score of EMSE in non-survivors was 22 points for EMSE-EAC and 44 points for EMSE-EACE.

### Prediction of functional outcome at discharge based on the EMSE and STESS

**Fig. 4:**
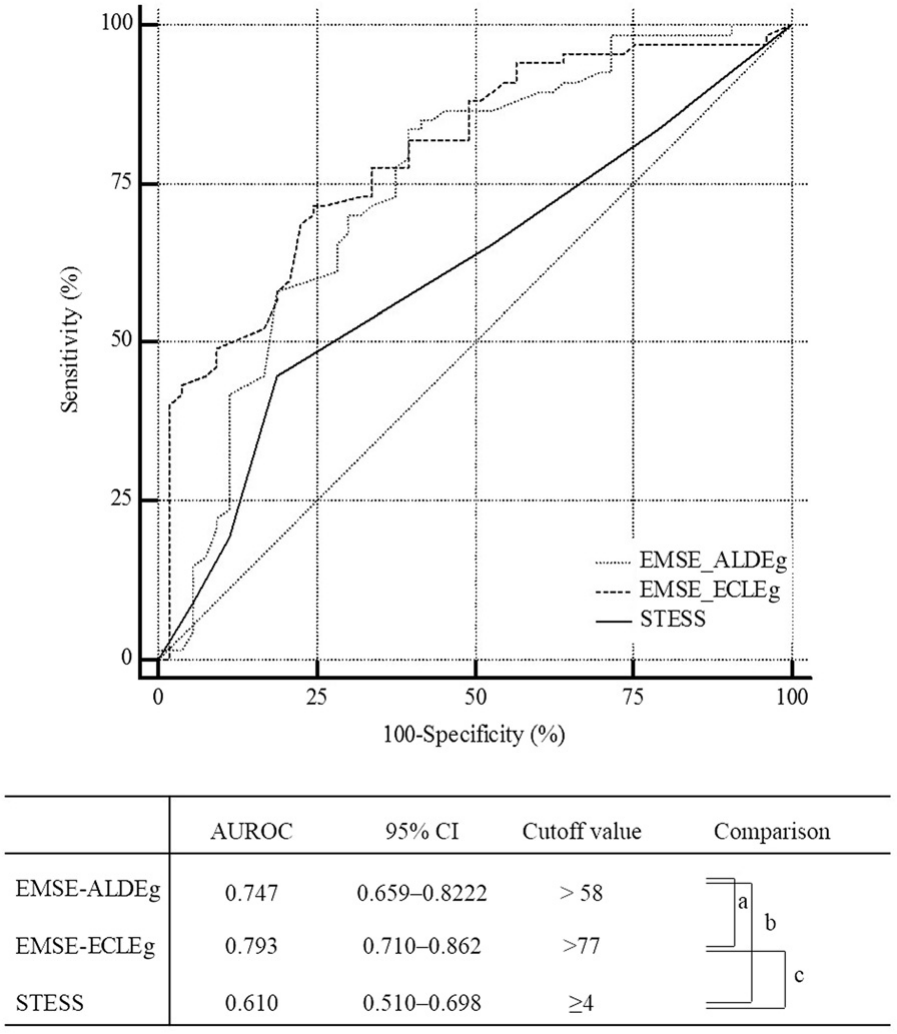
Receiver-operating characteristic curves for EMSE-ALDEg, EMSE-ECLEg and STESS for predicting poor functional outcome at discharge. Comparison *P* value: a = 0.1427, b = 0.0180, c = 0.0014. *AUROC* area under a receiver-operating characteristic curve, *CI* confidence interval, *EMSE* epidemiology-based mortality score in status epilepticus (E, etiology; A, age; C, comorbidity; L, level of consciousness at pretreatment; D, duration; Eg, electroencephalography), *STESS* status epilepticus severity score

All eight EMSE combinations that had the highest score for predicting in-hospital mortality had an AUC >0.740 for predicting the functional outcome (Table 3). Six of the eight EMSE combinations had the same or a higher calculated cutoff value for predicting the functional outcome compared with those for predicting in-hospital mortality. The ROC for predicting the functional outcome based on the EMSE-ALDEg and ECLEg had an AUC of 0.747 and 0.793, respectively, with an optimal cutoff value for discrimination (best match for both sensitivity (0.73 and 0.72, respectively) and specificity (0.74 and 0.75, respectively)) that was >58 and 77 points, respectively. The ROC for predicting the functional outcome based on the STESS had an AUC of 0.610, which was statistically inferior to that obtained based on the EMSE (Fig. 4).

**Table 3:** Quantitative description of the choice of EMSE combinations and STESS for predicting functional outcomes

	AUROC (cutoff point)	Sensitivity	Specificity	NPV	PPV	Accuracy
EMSE-ECEg	0.777 (>51)	0.82	0.60	0.73	0.72	0.73
EMSE-ECLEg	0.793 (>77)	0.72	0.75	0.68	0.79	0.73
EMSE-ALDEg	0.747 (>58)	0.73	0.74	0.60	0.84	0.73
STESS	0.610 (≥4)	0.81	0.44	0.54	0.75	0.61

*AUROC* area under a receiver-operating characteristic curve, *EMSE* epidemiology-based mortality score in status epilepticus (E, etiology; A, age; C, comorbidity; L, level of consciousness at pretreatment; D, duration; Eg, electroencephalography), *NPV* negative predictive value, *PPV* positive predictive value, *STESS* status epilepticus severity score

## Discussion

This was the first independent, external, head-to-head validation study of the predictive accuracy of two clinical scoring systems (STESS and EMSE) that are used for predicting in-hospital mortality among patients with SE. We also studied the potential role of these two scoring systems in predicting the functional outcome in these patients.

### Prediction of in-hospital mortality

We found that an EMSE-ALDEg (>60, age–loss of consciousness–duration–EEG) or an EMSE-ECLEg (>81, etiology–comorbidity–loss of consciousness–EEG) was the best combination for predicting in-hospital mortality. At these cutoff points for the EMSE-ALDEg and EMSE-ECLEg, the NPV was 44.5 % and 95.6 % and the PPV was 100 % and 25.5 %, respectively. A previous study reported that a different combination of EMSE parameters, EAC (etiology–age–comorbidity) and EACE (etiology–age–comorbidity–EEG), was the best combination to explain individual mortality, with 100 % NPV and 68.8 % PPV [2]. Demographic and methodological differences may explain why the best EMSE combination was different and the PPV was lower compared with the previous study. The in-hospital mortality and the proportion of patients over age 65 were higher in the previous report [2] than they were in our study (23.9 % versus 13.3 % and 62.0 % versus 47.5 %, respectively). Our population had 64.2 % patients with pre-existing epilepsy compared to 50 % in the initial EMSE population. This impacts results as the ceiling effect of STESS vanishes with increasing percentage of pre-existing epilepsy and helps to explain the lack of significant differences in mortality prediction. Only 27.5 % of patients had no comorbidity in our study compared to 43.5 % in the initial EMSE population. The methods used to decide on the optimized cutoff value for the EMSE combination in our study were also different from those used in the previous one. The lowest total score on the EMSE combination in non-survivors was taken as the cutoff value to improve the PPV in the previous study, whereas the calculated cutoff value of the ROC was used in our study. Thus high EMSE score in our population could be the main reason for low-to-moderate performance of EMSE in predicting mortality. Ninety five patients (79.2 %) scored 33 points for seizure duration and 22 (19.2 %) and 65 (54.2 %) scored 60 and 50 points for EEG, respectively. Forty six patients (38.4 %) had 3 or more Charlson’s comorbidity index.

Here, the optimized cutoff value of the STESS was ≥4 (best match for both sensitivity (0.69) and specificity (0.41)), which was different from that proposed by the initial study (≥3 points) [3]. A recent independent external validation study of the STESS reported that a cutoff value of ≥4 points was optimal for predicting in-hospital death [4]. At this cutoff point, the NPV was 91.28 % and the PPV was 22.50 %. These results are very similar to those of prior studies of the STESS [3, 4]. There was no statistically significant difference between the predictive power of the STESS and the EMSE regarding in-hospital mortality.

### Prediction of functional outcome

In this study, an EMSE-ALDEg (>58) or an EMSE-ECLEg (>77) was the best combination for simultaneously predicting functional outcome at discharge and in-hospital death. In relation to prediction of the functional outcome, the EMSE-ALDEg and EMSE-ECLEg had an NPV of 60.4 % and 67.8 % and a PPV of 84.3 % and 78.7 %, respectively. This means that a positive test had a ~75 % chance of reflecting a bad outcome and a negative test had a ~70 % chance of reflecting a good outcome. Therefore, the EMSE-ALDEg and EMSE-ECLEg may be useful for simultaneously predicting survival and the functional outcome at discharge in patients with SE. In contrast, the STESS was significantly less useful in predicting the functional outcome.

In our study, less than half of the patients (56 out of 120, 47.7 %) were discharged with a good functional outcome. Although long-term outcome data were not collected in our study, the neurological function could be recovered after discharge. In a recent review of refractory or super-refractory SE, the long-term outcome was death (35 %), severe neurological deficit (13 %), mild neurological deficit (13 %), undefined deficit (4 %), and recovery to the baseline (35 %) among a total of 596 cases [9]. A prospective observational cohort study reported that 146 out of 248 patients (58.9 %) with convulsive SE achieved a good recovery (51 with GOS 4 and 95 with GOC 5) at 90 days after discharge from an intensive care unit [10]. Older age, refractory SE, a longer duration of seizure before treatment (over 1 hour), and the presence of burst suppression or periodic discharge at EEG were associated with a poor functional outcome at discharge in our study. A longer seizure duration, the presence of cerebral insult, and refractory convulsive SE were associated with a poor long-term functional outcome [10].

Maximum performance of the EMSE was achieved by incorporating EEG patterns categorized as lateralized or generalized periodic discharges. The presence of generalized periodic EEG discharges was a prognostic factor, with an odds ratio of 8.5 (95 % confidence interval 1.7–43.4; *P* = 0.01) for a poor outcome in a multivariate model [11]. Nei et al. reported that periodic discharges, either lateralized or generalized, are associated with a poor outcome in SE [12]. Refractory SE was defined as SE that was unresponsive to treatment with at least two or more antiepileptic drugs and is a well-known independent factor for outcome prediction [5]. Because patients with SE usually undergo their first EEG study after first- or second-line antiepileptic drug treatment in clinical practice, the EEG abnormalities observed in this study may in fact reflect a refractory SE. Ideally, the best scoring system in clinical practice should be available at the most relevant time such as entering hospital or diagnosing the SE. However, in many hospital dealing with SE, the EEG has been recorded hours to days later. It could be limited to apply the EMSE system in clinical practice.

### Strengths and limitations

The strengths of this study were the inclusion of a large multicenter prospective cohort and the fact that its investigators were independent from the research group that developed the two clinical scoring systems. Data collection regarding the integral variables of the EMSE and STESS was performed blindly to other clinical data to avoid investigator biases. The limitations of this study were the retrospective nature of the analysis from the dataset, because our cohort was not designed for this analysis. The Charlson’s comorbidity index and seizure duration were scored retrospectively. Our database could not represent the epidemiology of South Korea and it is hard to directly compare our result with original research on EMSE score because only 120 cases of SE were enrolled from eight hospitals (3–24 patients from each center) for 2 years in an area with an estimated 26 million inhabitants.

## Conclusion

This is the first independent, external, head-to-head validation study of the predictive accuracy of STESS and EMSE score using a multicenter prospective cohort. Our results suggest that there was no statistically significant difference between the predictive power of two scoring systems regarding in-hospital mortality. It is possible to predict functional outcome (EMSE-ADLEg-58 or EMSE-ECLEg-77) and in-hospital death (EMSE-ADLEg-60 or EMSE-ECLEg-81) using the EMSE score, simultaneously. However, the best combination of EMSE remained unclear. The optimized cutoff value of STESS could be at greater than or equal to 4 points to predict in-hospital death.

## Key messages

- This is the first independent, external, validation study of the predictive accuracy of STESS and EMSE score using a multicenter prospective cohort.
- There was no statistically significant difference between the predictive power of two scoring system regarding in-hospital mortality.
- It is possible to predict functional outcome and in-hospital death using the EMSE score, simultaneously.
- The STESS was significantly less useful in predicting the functional outcome.
- The optimized cutoff value of STESS could be at greater than or equal to 4 points to predict in-hospital death.

## Additional files

Additional file 1: **All other ethical bodies that approved our study in the various centers.** (DOCX 12 kb)

Additional file 2: Table S1. Receiver-operating characteristic curve analysis for predicting in-hospital death based on STESS and EMSE combinations. (DOCX 17 kb)

## Abbreviations

AUC: Area under the curve
AUROC: Area under a receiver-operating characteristic curve
EEG: Electroencephalography
EMSE: Epidemiology-based mortality score in status epilepticus
GCSE: Generalized convulsive status epilepticus
GOS: Glasgow Outcome Scale
LOC: Level of consciousness
NCSE: Non-convulsive status epilepticus
NPV: Negative predictive value
PPV: Positive predictive value
ROC: Receiver-operating characteristic
SE: Status epilepticus
STESS: Status epilepticus severity score
