# Supplementary material for: Prediction of mortality and functional outcome from status epilepticus and independent external validation of STESS and EMSE scores
Source: Crit Care. 2016 Jan 27;20:25. doi: 10.1186/s13054-016-1190-z (PMC4728818; doi:10.1186/s13054-016-1190-z)
Supplement: Supplementary file 1 — All other ethical bodies that approved our study in the various centers. (DOCX 12 kb) [file 13054_2016_1190_MOESM1_ESM.docx]

**All other ethical bodies that approved our study in the various centers involved:**

The institutional review board of Konkuk University Medical Center.

The institutional review board of Dongguk University Ilsan Hostpial.

The institutional review board of Keimyung University Dongsan Medical Center

The institutional review board of Samsung Changwon Hospital

The institutional review board of National Medical Center

The institutional review board of Kangwon National University Hospital

The institutional review board of Seoul National University Hostpial, Biomedical Research Institute
