# Supplementary material for: Prediction of mortality and functional outcome from status epilepticus and independent external validation of STESS and EMSE scores
Source: Crit Care. 2016 Jan 27;20:25. doi: 10.1186/s13054-016-1190-z (PMC4728818; doi:10.1186/s13054-016-1190-z)
Supplement: Supplementary file 2 — Receiver-operating characteristic curve analysis for predicting in-hospital death based on STESS and EMSE combinations. (DOCX 17 kb) [file 13054_2016_1190_MOESM2_ESM.docx]

Additional file 2: Table S1. Receiver-operating characteristic curve analysis for predicting in-hospital death based on STESS and EMSE combinations.

|  | **Etiology (E)** | **Age (A)** | **Comorbidity (C)** | **LOC (L)** | **Duration (D)** | **EEG (E)** |
| --- | --- | --- | --- | --- | --- | --- |
|  | 0.661 | 0.611 | 0.583 | 0.657 | 0.584 | 0.671 |
| E |  | 0.681 | 0.617 | 0.691 | 0.675 | 0.721 |
| A |  |  | 0.596 | 0.693 | 0.657 | 0.695 |
| C |  |  |  | 0.630 | 0.624 | 0.697 |
| L |  |  |  |  | 0.724 | 0.734 |
| D |  |  |  |  |  | 0.692 |
| EA |  |  | 0.633 | 0.712 | 0.685 | 0.724 |
| EC |  |  |  | 0.664 | 0.647 | **0.750** |
| EL |  |  |  |  | 0.718 | **0.764** |
| ED |  |  |  |  |  | 0.725 |
| AC |  |  |  | 0.641 | 0.635 | 0.703 |
| AL |  |  |  |  | 0.736 | 0.739 |
| AD |  |  |  |  |  | 0.708 |
| CL |  |  |  |  | 0.665 | 0.718 |
| CD |  |  |  |  |  | 0.700 |
| LD |  |  |  |  |  | **0.744** |
| EAC |  |  |  | 0.676 | 0.657 | **0.712** |
| EAL |  |  |  |  | 0.728 | **0.771** |
| EAD |  |  |  |  |  | 0.727 |
| ECL |  |  |  |  | 0.687 | **0.745** |
| ECD |  |  |  |  |  | 0.703 |
| ELD |  |  |  |  |  | **0.766** |
| ACL |  |  |  |  | 0.673 | 0.728 |
| ACD |  |  |  |  |  | 0.704 |
| ALD |  |  |  |  |  | **0.745** |
| CLD |  |  |  |  |  | 0.722 |
| STESS | AUROC = 0.673 | | P = 0.0204 | Cutoff ≥4 |  |  |
